# Continuous Stakeholder Engagement: Expanding the Role of Pharmacists in Prevention of Type 2 Diabetes Through the National Diabetes Prevention Program

**DOI:** 10.5888/pcd17.190374

**Published:** 2020-06-04

**Authors:** Leslie W. Ross, Farida Bana, Rachel J. Blacher, Judith McDivitt, Joshua Petty, John Beckner, Robert Montierth

**Affiliations:** 1 Division of Diabetes Translation, National Center for Chronic Disease Prevention and Health Promotion, Centers for Disease Control and Prevention, Atlanta, Georgia; 2 Oak Ridge Institute for Science and Education, Oak Ridge, Tennessee; 3 CDC Foundation, Atlanta, Georgia; 4 National Community Pharmacists Association, Alexandria, Virginia

## Abstract

The pharmacy sector is a key partner in the National Diabetes Prevention Program (National DPP), as pharmacists frequently care for patients at high risk for type 2 diabetes. The Centers for Disease Control and Prevention aimed to increase pharmacist involvement in the program by leveraging partnerships with national pharmacy stakeholders. Continuous stakeholder engagement helped us to better understand the pharmacy sector and its needs. With stakeholders, we developed a guide and promotional campaign. By following a systematic process and including key stakeholders at every step of development, we successfully engaged these valuable partners in national type 2 diabetes prevention efforts. More pharmacy sites (n = 87) are now offering the National DPP lifestyle change program compared to before release of the guide (n = 27).

**Summary**

**What is already known on this topic?**

Pharmacists are well positioned and can be highly effective in providing preventive health services to patients in their communities; however, they remain underutilized as public health service providers.

**What is added by this report?**

We strategically leveraged partnerships with pharmacy stakeholders to develop resources and promotional materials tailored to the needs and values of pharmacists. Our efforts can help expand type 2 diabetes prevention services through the pharmacy workforce.

**What are the implications for public health practice?**

A systematic process of continuous stakeholder engagement can be replicated as an approach to involve pharmacists and other health professionals in similar public health prevention efforts.

## Background

One in 3 US adults has prediabetes, which can lead to type 2 diabetes, heart disease, and stroke (1). To help prevent or delay type 2 diabetes, the Centers for Disease Control and Prevention (CDC) established the National Diabetes Prevention Program (National DPP) in 2010. The National DPP is a partnership of public and private organizations building a nationwide delivery system for an evidence-based lifestyle change program for adults at high risk for type 2 diabetes. The program follows a CDC-approved curriculum and is delivered by trained lifestyle coaches in person or virtually. The goal is to help participants engage in healthy behaviors and achieve 5% to 7% weight loss (2). Evidence shows that participants in the National DPP lifestyle change program can cut their risk of developing type 2 diabetes by 58% and 71% for people aged 60 years or older (3). The National DPP lifestyle change program is offered in various settings, such as hospitals and clinics, community organizations, and worksites.

Pharmacists are the third largest group of health care professionals in the United States, after physicians and nurses, and are often on the front lines of care for medically underserved patients who are at risk for type 2 diabetes. Despite the extensive training of pharmacists and their service expansion beyond traditional medication dispensing, they remain underused as public health service providers (4). Ninety-two percent of US residents live within 1.6 miles of a pharmacy, and patients see their pharmacist more frequently than their primary care physician (5,6). Pharmacies are well positioned to provide preventive health services because of their convenient locations and extended hours of operation, which allow them to reach patients who might otherwise have limited access to care (5). They often provide services that align with National DPP activities, including patient education, screening and identifying patients at high risk for chronic disease, initiating referrals, and providing chronic disease and weight management services (5,7,8). 

## Intervention Approach

CDC manages quality assurance of the National DPP, awarding CDC recognition to organizations that deliver the lifestyle change program and meet national quality standards (9). In 2016, CDC identified 7 pharmacies already offering the lifestyle change program and invested in efforts to determine how the pharmacy workforce (pharmacists, technicians, residents, community health workers, and students) could expand and sustain the National DPP. This CDC multiyear effort was to scale the National DPP in more pharmacies by collaborating with national pharmacy stakeholders and leveraging these partnerships to better understand the pharmacy landscape, develop a tailored resource for pharmacists, and disseminate pharmacy-specific information about the National DPP to the pharmacy workforce (Figure).

**Figure:**
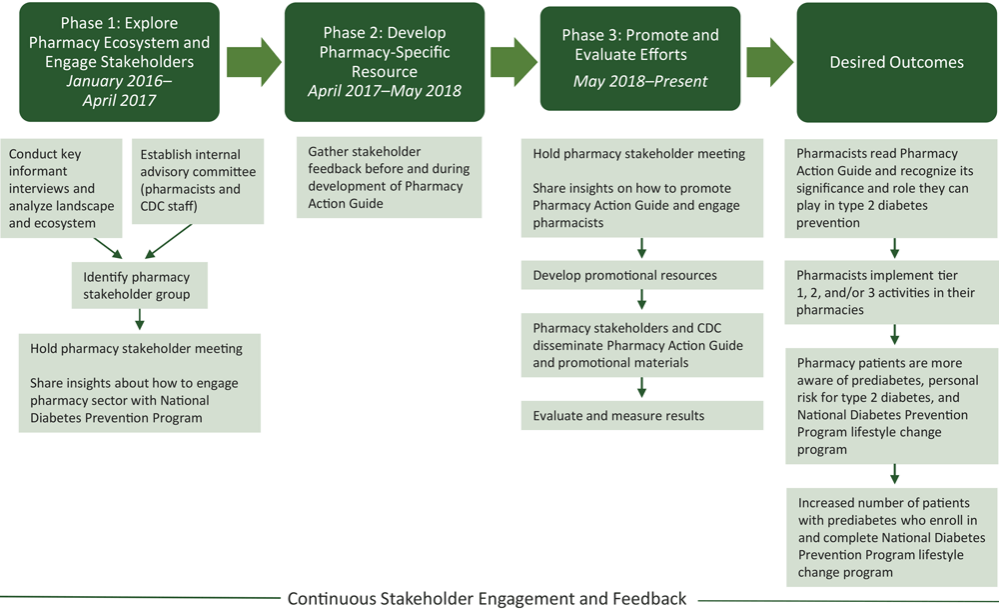
Model for pharmacist engagement in the National Diabetes Prevention Program.

### Phase 1: explore and engage

We began by establishing relationships with the 7 pharmacies already implementing the National DPP lifestyle change program and exploring their motives for involvement. These pharmacies serve diverse patient populations across the United States. Five serve rural populations, and 4 focus on patients who have a low income or who are medically underserved. Five are independent pharmacies; 1 is a school of pharmacy; and 1 is a retail chain grocery pharmacy. We learned through key informant interviews that several of these pharmacies attributed success in implementing the National DPP to alignment of the program with wellness services the pharmacies already offered, sufficient financial and staffing support, and strong preexisting relationships in their communities. Our pharmacy ecosystem and landscape analysis indicated the following facts:

- Pharmacists are increasingly working as providers, expanding their portfolio of patient care services.
- More independent pharmacies than other pharmacy types exist in areas with a high prevalence of diabetes, although their limited resource capacity makes scalability a challenge.
- National pharmacy associations are strong advocates for enabling pharmacists to offer more services and can serve as influencers in pharmacist decision making.

Based on results of the analysis and recommendations from an internal advisory committee, we established a national pharmacy stakeholder group of pharmacy associations, pharmacies implementing or interested in the National DPP lifestyle change program, and public health and pharmacy representatives from government and advocacy groups. We involved this group in decisions and strategies to engage the pharmacy sector in National DPP activities.

In March 2017, we convened a meeting of pharmacy stakeholders (Box). Representatives of 10 national pharmacy organizations attended to discuss pharmacy roles in the National DPP, marketing and communication strategies, and resources needed for engagement. Stakeholders shared insights on the validity of the landscape analysis, determined how the National DPP could fit within the pharmacy sector, and stressed the need for resources, including concrete, practical information on how pharmacies could become involved in type 2 diabetes prevention efforts. They shared ideas for content for a guidance document, including how pharmacies can incorporate the program into their workflows.

**Box. Organizations Represented at Pharmacy Stakeholder Meetings, 2017–2018**

**Pharmacies and Pharmacy Associations**

American Association of Colleges of Pharmacy
American Pharmacists Association
American Society of Health-System Pharmacists
CVS Health — 2nd meeting only, May 24, 2018
Georgia Community Pharmacy Enhanced Services Network
National Alliance of State Pharmacy Associations
National Association of Chain Drug Stores
National Community Pharmacists Association

**Pharmacies Delivering the National Diabetes Prevention Program Lifestyle Change Program**

Duquesne University Center for Pharmacy Care
Jefferson Community Health and Life
Kroger, Kentucky (Louisville Division)

**Federal Government: Public Health and Pharmacist Representatives**

Center for Medicare and Medicaid Innovation, Centers for Medicare & Medicaid Services — 2nd meeting only, May 24, 2018
Centers for Disease Control and Prevention
United States Department of Veterans Affairs — 2nd meeting only, May 24, 2018
United States Public Health Service — 2nd meeting only, May 24, 2018

**Public Health Advocacy**

American Diabetes Association — 2nd meeting only, May 24, 2018

### Phase 2: develop and test

In Phase 2, we collaborated with pharmacy stakeholders to develop and test a resource tailored to the needs of pharmacists that would prompt their involvement in the National DPP. Stakeholders provided instrumental insights for the development of the Rx for the National Diabetes Prevention Program: Action Guide for Community Pharmacists (Pharmacy Action Guide), released in May 2018. At the March 2017 meeting, stakeholders conveyed that, as a result of limited time and resources, not all pharmacies would be able to deliver the lifestyle change program, but they could support the National DPP in other ways. Therefore, the guide highlighted the following 3 tiers of pharmacy involvement:

- Tier 1: Promote awareness of prediabetes and the National DPP. This is a simple, low-cost step to get involved with type 2 diabetes prevention efforts. The guide outlines existing promotional materials and campaigns that pharmacies can use to raise awareness of prediabetes and the National DPP.
- Tier 2: Screen, test, and refer. By using a CDC-approved risk assessment or blood glucose test, pharmacies can help determine whether patients are at high risk of developing type 2 diabetes or currently have prediabetes. Pharmacy staff can then refer eligible patients to the CDC-recognized lifestyle change program.
- Tier 3: Offer the lifestyle change program. The guide describes how pharmacies can become CDC-recognized providers of the National DPP lifestyle change program.

Based on stakeholder feedback on the capacity of pharmacists versus other pharmacy staff members (residents, students, technicians) to implement the 3 tiers, the guide outlines how members of the pharmacy workforce can become involved in expanding the reach of the National DPP. Stakeholders from a university and retail grocery pharmacy shared case studies describing how they operationalized tiers 2 and 3 in their pharmacy settings, respectively (10).

Furthermore, stakeholders provided feedback on multiple iterations of the guide. Some also shared copies of the draft guide with pharmacists in their networks, who provided additional feedback that we incorporated. This iterative process of incorporating revisions from our target audience resulted in a comprehensive resource, providing user-friendly, motivational information specific to the needs of the pharmacy sector.

### Phase 3: promote and evaluate

The purpose of Phase 3 was to strengthen partnerships within the pharmacy sector, create a campaign to promote the Pharmacy Action Guide, increase pharmacist awareness and uptake of National DPP activities, and evaluate efforts. We developed the promotion campaign on the basis of what we learned about the pharmacy landscape through our partners. Furthermore, we leveraged our partnerships to disseminate the guide and accompanying promotional materials.

After release of the Pharmacy Action Guide, we facilitated a second stakeholder meeting in May 2018. Attendees shared ideas about motivating pharmacists to read and adopt the guide, supplemental resources for pharmacist decision making about adopting the 3 tiers, and strategic communication channels. Stakeholders also discussed potential barriers and facilitators to success in pharmacist engagement that we incorporated into a promotion framework.

We tailored promotional messages to align with pharmacists’ values. Stakeholders noted that many pharmacists are motivated by a sense of commitment to community health but need a business case to ensure National DPP efforts are feasible and sustainable. Based on these insights, we chose 2 key messages as a focus for the first phase of the promotion campaign.

- Engagement in National DPP activities provides an opportunity to diversify pharmacy services and revenue in an increasingly competitive market.
- Offering National DPP-related services is a way to help the community by improving patient outcomes, while reinforcing perceptions of pharmacists as trusted sources for preventive care.

We used testimonials from pharmacy program advocates to show pharmacists that their peers were implementing activities successfully. Given that pharmacy associations are key influencers for the pharmacy audience, they are critical partners to establish pharmacist buy-in. Pharmacy association stakeholders disseminated key messages to their members with links to the guide and additional resources. They reached pharmacists at multiple touchpoints through a multichannel marketing and outreach strategy that combined traditional (direct mail, event marketing) and digital media (email, web, social media, video marketing). 

We gained momentum by strategically targeting segments of the pharmacy community most likely to adopt type 2 diabetes prevention activities. We started with independent and grocery retail pharmacists, because many successful early implementers of the program belonged to these 2 groups. Their values and locations were also well-suited to offer type 2 diabetes prevention services in communities at high risk.

We are evaluating our promotion efforts to measure pharmacy uptake of National DPP activities and will use results to prioritize strategies for future promotion. Early results are encouraging. In June 2019, we provided marketing toolkits to 12 pharmacy associations. Of those, 7 have disseminated promotional materials to their members, resulting in more than 2,100 video views and 4,200 downloads of the Pharmacy Action Guide as of October 2019. In addition, significant growth occurred in the number of pharmacies seeking CDC recognition to offer the National DPP lifestyle change program. In October 2019, 87 pharmacy organizations had CDC recognition, many in underserved areas, compared with 27 before release of the guide.

## Implications for Public Health Practice

Although we attempted to create accessible resources relevant to the entire pharmacy workforce, limitations existed for what we could accomplish in a national engagement campaign. Differences exist state-to-state regarding pharmacist scope of practice (eg, blood glucose testing), and each pharmacy has its own unique facilitators and barriers to engage in type 2 diabetes prevention activities. Although CDC might not be able to address the myriad needs of the entire pharmacy workforce related to National DPP adoption, we are more in tune and better positioned to support our partners as they expand the reach of our efforts.

Our successes in collaborating with pharmacists in the National DPP demonstrate how partnerships between public health and the pharmacy sector can expand and sustain prevention efforts. Pharmacists are accessible, credible, and dedicated health professionals who can play a key role in preventing type 2 diabetes and other chronic diseases while addressing health inequities in their communities. Our project sought to establish reciprocal support and feedback between the public health and pharmacy sectors. Through continual stakeholder engagement and inclusion of members of the pharmacy community at every step, we collaboratively built a relevant and successful program that optimized pharmacist involvement in national type 2 diabetes prevention efforts. This systematic process of stakeholder engagement and iteration can be replicated as a model for engaging pharmacists and other health professionals in similar public health prevention efforts. Information on how pharmacists can become involved in the National DPP is available at https://www.cdc.gov/diabetes/prevention/pharmacists.html.

## References

[1] Centers for Disease Control and Prevention. National diabetes statistics report, 2017: estimates of diabetes and its burden in the United States. https://www.cdc.gov/diabetes/pdfs/data/statistics/national-diabetes-statistics-report.pdf. Accessed August 9, 2019.

[2] Centers for Disease Control and Prevention. About the National DPP. https://www.cdc.gov/diabetes/prevention/about.htm. Accessed August 9, 2019.

[3] Diabetes Prevention Program Research Group. Reduction in the incidence of type 2 diabetes with lifestyle intervention or metformin. N Engl J Med 2002;346(6):393–403. 10.1056/NEJMoa012512 11832527 PMC1370926

[4] Mossialos E , Courtin E , Naci H , Benrimoj S , Bouvy M , Farris K , From “retailers” to health care providers: transforming the role of community pharmacists in chronic disease management. Health Policy 2015;119(5):628–39. 10.1016/j.healthpol.2015.02.007 25747809

[5] San-Juan-Rodriguez A , Newman TV , Hernandez I , Swart ECS , Klein-Fedyshin M , Shrank WH , . Impact of community pharmacist-provided preventive services on clinical, utilization, and economic outcomes: an umbrella review. Prev Med 2018;115:145–55. 10.1016/j.ypmed.2018.08.029 30145351

[6] Feehan M , Walsh M , Godin J , Sundwall D , Munger MA . Patient preferences for healthcare delivery through community pharmacy settings in the USA: a discrete choice study. J Clin Pharm Ther 2017;42(6):738–49. 10.1111/jcpt.12574 28627110

[7] Kelling SE , Rondon-Begazo A , DiPietro Mager NA , Murphy BL , Bright DR . Provision of clinical preventive services by community pharmacists. Prev Chronic Dis 2016;13: 160232. 10.5888/pcd13.160232 27788064 PMC5084625

[8] Brown TJ , Todd A , O’Malley C , Moore HJ , Husband AK , Bambra C , Community pharmacy-delivered interventions for public health priorities: a systematic review of interventions for alcohol reduction, smoking cessation and weight management, including meta-analysis for smoking cessation. BMJ Open 2016;6(2):e009828. 10.1136/bmjopen-2015-009828 26928025 PMC4780058

[9] Centers for Disease Control and Prevention. Centers for Disease Control and Prevention Diabetes Prevention Recognition Program standards and operating procedures 2019. https://www.cdc.gov/diabetes/prevention/pdf/dprp-standards.pdf. Accessed August 20, 2019.

[10] Centers for Disease Control and Prevention. Rx for the National Diabetes Prevention Program: action guide for community pharmacists. https://www.cdc.gov/diabetes/prevention/pdf/pharmacists-guide.pdf. Accessed August 9, 2019.

